# Guanylate cyclase-C/cGMP: an emerging pathway in the regulation of visceral pain

**DOI:** 10.3389/fnmol.2014.00031

**Published:** 2014-04-16

**Authors:** Gerhard Hannig, Boris Tchernychev, Caroline B. Kurtz, Alexander P. Bryant, Mark G. Currie, Inmaculada Silos-Santiago

**Affiliations:** Department of Discovery Pharmacology, Ironwood Pharmaceuticals, Inc., CambridgeMA, USA

**Keywords:** abdominal pain, colonic nociceptor, cyclic guanosine monophosphate, guanylate cyclase-C, irritable bowel syndrome with constipation, linaclotide, uroguanylin, visceral analgesia

## Abstract

Activation of guanylate cyclase-C (GC-C) expressed predominantly on intestinal epithelial cells by guanylin, uroguanylin or the closely related GC-C agonist peptide, linaclotide, stimulates generation, and release of cyclic guanosine-3′,5′-monophosphate (cGMP). Evidence that the visceral analgesic effects of linaclotide are mediated by a novel, GC-C-dependent peripheral sensory mechanism was first demonstrated in animal models of visceral pain. Subsequent studies with uroguanylin or linaclotide have confirmed the activation of a GC-C/cGMP pathway leading to increased submucosal cGMP mediated by cGMP efflux pumps, which modulates intestinal nociceptor function resulting in peripheral analgesia. These effects can be reproduced by the addition of exogenous cGMP and support a role for GC-C/cGMP signaling in the regulation of visceral sensation, a physiological function that has not previously been linked to the GC-C/cGMP pathway. Notably, targeting the GC-C/cGMP pathway for treatment of gastrointestinal pain and abdominal sensory symptoms has now been validated in the clinic. In 2012, linaclotide was approved in the United States and European Union for the treatment of adult patients with irritable bowel syndrome with constipation.

## INTRODUCTION

Guanylate cyclase-C (GC-C) is a type I transmembrane receptor with intrinsic guanylate cyclase activity, belonging to a larger family of enzymes comprising both soluble and receptor guanylate cyclases that respond to a diverse range of signals by catalyzing the conversion of guanosine triphosphate to cyclic guanosine-3′,5′ monophosphate (cGMP; Lucas et al., 2000; Vaandrager, 2002). This receptor is expressed predominantly on the apical surface of intestinal epithelial cells (IEC), and its important role in the regulation of intestinal electrolyte and fluid homeostasis is firmly established (Schulz et al., 1990; Carrithers et al., 1996; Castro et al., 2013; Silos-Santiago et al., 2013). The endogenous hormones guanylin and uroguanylin, cognate ligands of GC-C, are expressed in a distinct but overlapping regional pattern in enteroendocrine cells along the longitudinal axis of the gastrointestinal (GI) tract and released into the intestinal lumen (Currie et al., 1992; Kita et al., 1994; Perkins et al., 1997; Qian et al., 2000; Silos-Santiago et al., 2013). Activation of GC-C by either guanylin or uroguanylin results in cGMP accumulation in IEC. Cyclic GMP is the sole second messenger generated by GC-C and is known to regulate several canonical intracellular signaling pathways mediated primarily through direct interaction with three groups of target proteins: cGMP-dependent protein kinases, cyclic nucleotide-gated ion channels, and cGMP-regulated phosphodiesterases (Pfeifer et al., 1996; Schlossman et al., 2005). Cyclic GMP regulation of intestinal electrolyte and fluid homeostasis is dependent on protein kinase II (PKG-II) phosphorylation and activation of the cystic fibrosis transmembrane conductance regulator (CFTR) ion channel, stimulating transepithelial secretion of chloride and bicarbonate ions and concomitant inhibition of sodium absorption by the sodium/hydrogen exchanger 3 (NHE3), resulting in the net efflux of ions and water into the lumen (Forte, 1999; Vaandrager, 2002; Sindic and Schlatter, 2006). Studies in GC-C null (*Gucy2c*^-^^/^^-^) mice have confirmed the central role of GC-C in the control of electrolyte and fluid homeostasis (Mann et al., 1997; Schulz et al., 1997).

Our understanding of cellular processes regulated by activation of the GC-C/cGMP pathway has markedly evolved over the past two decades. Importantly, this knowledge has guided the development of novel therapeutic paradigms targeting the GC-C/cGMP pathway that have successfully translated into the clinic.

## LINACLOTIDE IS A POTENT AND SELECTIVE GUANYLATE CYCLASE-C AGONIST

Linaclotide, a synthetic 14-amino acid peptide composed of naturally occurring amino acids (N-CCEYCCNPACTGCY-C; molecular weight: 1524.2 Dalton), is a potent and selective agonist of GC-C. This orally administered, minimally absorbed peptide (≤0.1% in all non-clinical species) is a member of the guanylin family of cGMP-regulating peptides that includes the natural hormones guanylin and uroguanylin (Bryant et al., 2010; Busby et al., 2010). Linaclotide is stabilized by three intramolecular disulfide bonds (Cys^1^-Cys^6^, Cys^2^-Cys^10^, Cys^5^-Cys^13^), locking this peptide into a single conformation similar in structure to guanylin and uroguanylin, with the distinct difference that the biologically active conformation (A-isoform) of these peptides contains only two disulfide bonds (Skelton et al., 1994; Marx et al., 1998; Busby et al., 2010). Linaclotide exhibited high-affinity and pH-independent binding to GC-C on human colon carcinoma T84 cells and concomitantly stimulated a significant and concentration-dependent accumulation of intracellular cGMP, with greater potency than guanylin or uroguanylin. In standard rodent models of GI function, orally administered linaclotide elicited significant *in vivo* pharmacological effects, stimulating fluid secretion and accelerating transit (Bryant et al., 2010; Busby et al., 2010). The lack of such effects in *Gucy2c*^-^^/^^-^mice further confirmed GC-C as the molecular target of linaclotide and supported an underlying mechanism linking the effects of linaclotide in these models to local activation of GC-C in the intestine. The single pharmacologically active metabolite of linaclotide, MM-419447 (Des-Tyr^14^) mirrors the effects of linaclotide *in vitro* and *in vivo*, providing evidence that this active metabolite contributes to the pharmacology associated with oral administration of linaclotide (Busby et al., 2013).

The potent pharmacological effects of linaclotide in non-clinical models provided the rationale for development of this peptide as a novel therapeutic for the treatment of functional GI disorders associated with constipation, such as chronic idiopathic constipation (CIC), and irritable bowel syndrome with constipation (IBS-C). However, no reports had previously linked local activation of the intestinal GC-C pathway to the regulation of abdominal pain.

## LINACLOTIDE ACTIVATION OF GUANYLATE CYCLASE-C ELICITS ANALGESIC EFFECTS IN VISCERAL PAIN MODELS

Abdominal pain or discomfort (associated with a change in bowel function) is a defining symptom of the diagnostic criteria for IBS and is hypothesized to originate from hypersensitivity of the colon to mechanical stimuli (Drossman, 2006; Tack et al., 2006; Videlock and Chang, 2007). In clinical studies, IBS patients have shown lowered colorectal pain thresholds and increased sensory ratings, further consistent with persistent enhanced perception and responsiveness to visceral stimuli (= visceral hyperalgesia; Mertz et al., 1995; Bouin et al., 2002).

Antinociceptive effects of an orally administered GC-C agonist, linaclotide, were first demonstrated by Eutamene et al. (2010), using rodent models of visceral hypersensitivity associated with inflammation produced by trinitrobenzene sulfonic acid (TNBS) or stress models (following acute water avoidance or partial restraint) that induce this condition. In these models, visceral pain is measured using colorectal balloon distension. This well-characterized method evokes contractions of the abdominal musculature (visceromotor response) to graded distension pressures. It differs from measurements of visceral sensitivity in humans, which is primarily based on conscious perception of controlled colon distension. Orally administered linaclotide elicited significant analgesic effects during colonic distension in all models tested, without affecting basal sensitivity. Notably, the specificity of linaclotide-mediated analgesic effects was mechanistically linked to the activation of intestinal GC-C, confirmed in studies that assessed the effects of linaclotide on TNBS-induced colonic hypersensitivity in *Gucy2c* wild-type and *Gucy2c*^-^^/^^-^ mice. In these studies, linaclotide reversed colonic hypersensitivity only in *Gucy2c* wild-type, but not *Gucy2c*^-^^/^^-^ mice. The analgesic effects of linaclotide were also reproduced by the endogenous GC-C ligand, uroguanylin, a finding not previously attributed to the endogenous hormones. The analgesic effects of linaclotide and uroguanylin in these models were not associated with changes in colonic pressure-volume relationships, further suggesting that the antinociceptive effects are linked specifically to GC-C activation and are not associated with altered colonic compliance (Eutamene et al., 2010; Silos-Santiago et al., 2013). These findings raised the intriguing question of whether activation of an intestinal GC-C pathway had emerged as a novel physiological pathway regulating visceral pain.

## LINACLOTIDE INDUCES PERIPHERAL VISCERAL ANALGESIA BY ACTIVATION OF A GUANYLATE CYCLASE-C/EXTRACELLULAR cGMP PATHWAY

Hallmarks of IBS include allodynia (persistent response to normally non-noxious stimuli) and hyperalgesia (persistent enhanced perception and responsiveness to noxious stimuli) to mechanical stimuli within the intestine, mediated by chronic changes in afferent pathways (sensitization). High-threshold colonic nociceptive sensory afferents (mesenteric, serosal afferents) that only respond at sufficiently high levels to mechanical stimulation are predominantly found in the splanchnic nerve pathway, while low-threshold stretch receptors [muscular/mucosal (M/M), muscular afferents] are predominantly found in the pelvic pathway (Hughes et al., 2009; Feng and Gebhart, 2011; Blackshaw and Brierley, 2013).

In a mouse model of chronic post-inflammatory visceral hypersensitivity (CVH) in which colonic mechanical hyperalgesia and allodynia are evident long after resolution of TNBS-induced colitis, linaclotide, and uroguanylin reversed chronic mechanical hypersensitivity in CVH colonic high-threshold splanchnic serosal nociceptors, and also inhibited mechanical hypersensitivity of healthy nociceptors. Inhibition of CVH colonic nociceptors by either peptide was greater than their inhibition of control nociceptors from healthy animals, and linaclotide-induced inhibition was more potent than that produced by uroguanylin. Importantly, *in vitro* inhibition of colonic nociceptors correlated with *in vivo* findings in which linaclotide decreased the processing of noxious colorectal distension stimuli in the thoracolumbar spinal cord indicated by a lower number of activated dorsal horn (DH) neurons within the thoracolumbar spinal cord, specifically the superficial lamina of the DH, recognized as the major termination zone for nociceptive afferents (Castro et al., 2013). Further evidence supporting a mechanism in which linaclotide inhibition of colonic nociceptors is dependent on local activation of GC-C in IEC rather than direct effects on colonic nociceptors was derived from expression studies using *in situ* hybridization in whole adult mouse, colonic segments, and spinal cord and dorsal root ganglion (DRG) sections, and studies assessing linaclotide inhibition of colonic nociceptors in *Gucy2c*^-^^/^^-^ mice. While abundant GC-C expression was found in the intestine, no expression was detected in key sensory structures, such as DRG and spinal cord neurons. Furthermore, while mechanosensory responses of colonic nociceptors at baseline were similar in control and *Gucy2*^-^^/^^-^ mice, linaclotide-induced inhibition was completely lost in *Gucy2c*^-^^/^^-^ mice. This was further corroborated by findings that prior removal of the mucosa, the source of GC-C, in healthy and CVH states significantly attenuated linaclotide inhibition of colonic nociceptors (Castro et al., 2013). Finally, electric field stimulation-induced contractions of colonic tissues were not affected by linaclotide, which confirmed that antinociceptive effects of linaclotide were not linked to altered smooth muscle cell contractility, consistent with the absence of GC-C expression on colonic smooth muscle cells (Castro et al., 2013).

While these findings indicate that the analgesic effects of linaclotide are mediated entirely by peripheral expression of GC-C on the apical surface of IEC, a direct inhibitory effect of linaclotide on colonic nociceptors was further considered unlikely because of minimal absorption of this peptide, and the location of peripheral endings of colonic nociceptors in the submucosa. This provoked studies that investigated the potential role of cGMP, the common primary downstream effector of linaclotide and uroguanylin, in altered intestinal nociceptor function and peripheral analgesia. The involvement of an intracellular GC-C/cGMP pathway in the regulation of intestinal fluid and electrolyte homeostasis is firmly established; however, the effects of extracellular cGMP transported out of IEC following local GC-C activation have remained elusive. Precedence that extracellular cGMP is involved in the modulation of neuronal activity was obtained from studies that showed direct inhibitory effects of extracellular cGMP on CNS neurons, resulting in reduced excitability and inhibition of neurotransmitter release (Linden et al., 1995; Poupoloupou and Nowak, 1998; Cervetto et al., 2010). When assessed in rat models of colonic hypersensitivity, orally administered cGMP elicited significant analgesic effects, in a dose-dependent manner, reproducing the effects of linaclotide and uroguanylin in these models (Silos-Santiago et al., 2013). There is now compelling evidence supporting a model in which the potent analgesic effects of cGMP *in vivo* are mediated by a pathway linking extracellular cGMP, secreted from IEC into the submucosa following activation of the GC-C/cGMP pathway by linaclotide or uroguanylin, to altered function of colonic nociceptors resulting in peripheral analgesia. *In vitro* exposure of human intestinal Caco-2 cells to linaclotide or uroguanylin stimulated extracellular transport of cGMP into the apical and basolateral spaces, which was inhibited by the cGMP efflux pump inhibitor probenecid in a concentration-dependent manner (Castro et al., 2013; Silos-Santiago et al., 2013). This provided evidence implicating energy-dependent transport of cGMP by the cGMP efflux pumps multidrug-resistance protein (MRP) 4 and 5 (Sager, 2004). While cGMP-binding phosphodiesterases are generally recognized as the major elimination pathway for intracellular cGMP, MRP4/5-mediated extracellular transport of cGMP is consistent with their function as overflow pumps, decreasing intracellular cGMP levels under conditions when cGMP production is strongly induced and importantly, providing extracellular cGMP for paracrine actions (Ritter et al., 2005; Zimmermann et al., 2005). Further evidence supporting a role of extracellular cGMP in the regulation of colonic afferent activity was obtained from *ex vivo* Ussing chamber assays, in which exposure of rat colonic tissue (luminal side) to uroguanylin stimulated secretion of cGMP into the submucosal space (Silos-Santiago et al., 2013). Moreover, in a rat model of TNBS-induced colonic afferent sensitization, exogenous cGMP significantly decreased pelvic afferent firing rates in response to colonic distension, and in CVH mice colonic nociceptors were significantly inhibited by application of exogenous cGMP to the mucosal epithelium, to a greater extent than those from healthy mice (Castro et al., 2013; Silos-Santiago et al., 2013). While cGMP dose levels required for inhibition of colonic nociceptors exceeded those for linaclotide and uroguanylin, facilitating access of cGMP to colonic nociceptors by removal of the mucosa significantly increased its potency, confirming the barrier function of the epithelium for luminal cGMP to diffuse across the mucosa (Castro et al., 2013). Furthermore, direct application of cGMP to mouse colorectal receptive endings significantly decreased the response of control pelvic muscular and M/M afferents to circumferential stretch, and sensitized responses of muscular and M/M afferents to stretch were reversed (Feng et al., 2013). Similar to findings with linaclotide and uroguanylin, antinociceptive effects of extracellular cGMP were not associated with altered smooth muscle contractility (Castro et al., 2013; Silos-Santiago et al., 2013).

**FIGURE 1:**
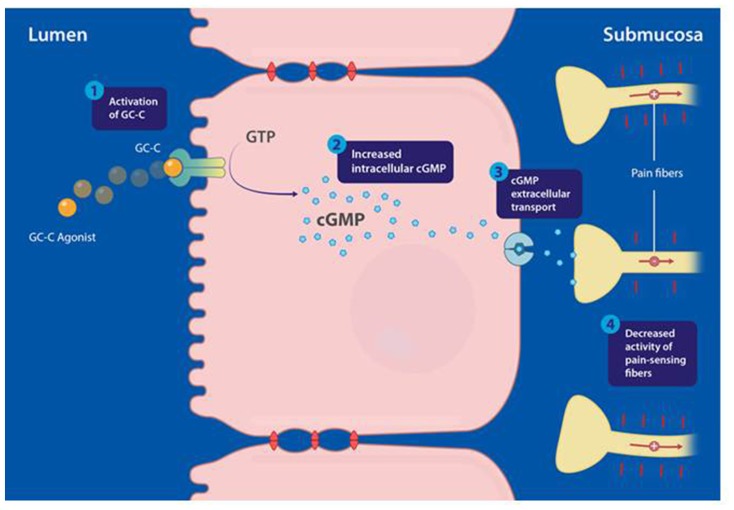
**Proposed mechanism of action of guanylate cyclase-C (GC-C) agonists modulating visceral pain, mediated through activation of the GC-C/cyclic guanosine-3′, 5′-monophosphate (cGMP) pathway**. (1) Linaclotide binds and activates GC-C, expressed at the apical surface of intestinal epithelial cells. (2) Activation of GC-C results in hydrolysis of guanosine triphosphate (GTP) and production of cGMP inside intestinal epithelial cells. (3) Intracellular cGMP is actively transported out of intestinal epithelial cells by efflux pumps into the submucosa. (4) Extracellular cGMP is proposed to inhibit colonic nociceptors. Adapted from Silos-Santiago et al. (2013); the figure has been reproduced with permission of the International Association for the Study of Pain^®^ (IASP).

In conclusion, accumulating evidence now strongly supports a direct peripherally acting analgesic mechanism that, following activation of a GC-C/extracellular cGMP pathway by selective GC-C agonists, mediates inhibition of colonic nociception and decreases visceral pain (**Figure 1**). This mechanism suggests that the regulation of colonic sensation may have evolved as an effect of GC-C agonism by the endogenous hormones guanylin and uroguanylin in IEC (Castro et al., 2013; Silos-Santiago et al., 2013).

## LINACLOTIDE DECREASES ABDOMINAL PAIN AND DISCOMFORT IN PATIENTS WITH IRRITABLE BOWEL SYNDROME WITH CONSTIPATION

Results from two pivotal phase 3 clinical trials with linaclotide in adult patients with IBS-C have validated the approach of therapeutically targeting the GC-C/cGMP pathway for treatment of abdominal pain in this disorder (Chey et al., 2012; Rao et al., 2012). The efficacy and safety of linaclotide were assessed in a 26 week, double-blind, parallel-group, placebo-controlled, randomized trial, and a 12 week, double-blind, parallel-group, placebo-controlled, randomized trial, with a 4 week randomized withdrawal period. In both trials, four prespecified primary endpoints were evaluated, based on the FDA primary endpoint for IBS-C [responder: improvement ≥30% from baseline in average daily worst abdominal pain score and increase by ≥1 complete spontaneous bowel movement (CSBM) from baseline (same week) for at least 50% of weeks assessed], and three other primary endpoints, based on improvements in abdominal pain and CSBM for 9/12 weeks. Notably, a 30% reduction in the pain score has previously been shown of clinical importance in IBS patients and in patients reporting pain relief in general (Farrar et al., 2001; Spiegel et al., 2009).

In the 26 week trial, 804 IBS-C patients randomized (1:1) to linaclotide or placebo, received oral linaclotide (290 μg, once daily) during the 26 week treatment period (primary and secondary efficacy assessments were evaluated over the first 12 weeks of treatment; Chey et al., 2012). For the rigorous, combined IBS-C endpoint recommended in the recent *Guidance for IBS Clinical Trials* (Food and Drug Administration, 2010), 33.7% of IBS-C patients in the linaclotide group were endpoint responders, compared with 13.9% in the placebo group (*P* < 0.0001). The FDA pain responder criterion was met by 48.9% of linaclotide-treated patients, compared to 34.5% in the placebo group (*P* < 0.0001) and similarly, 49.1% of linaclotide-treated patients met the abdominal pain endpoint for at least 13/26 weeks, compared to the placebo group (*P* < 0.0001). Furthermore, improvement in abdominal pain compared to placebo, was significant starting from the first week of therapy and continuing throughout the 26 week treatment period.

In the 12 week randomized trial, followed by a 4 week randomized withdrawal period, 800 patients (1:1, linaclotide or placebo) received oral linaclotide (290 μg, once daily) during the 12 week treatment period (Rao et al., 2012). Patients who had completed all 12 weeks of the double-blind treatment period were eligible to enter the double-blind 4 week randomized withdrawal period, in which patients initially randomized to linaclotide were re-randomized (1:1) to oral linaclotide (290 μg, once daily) or placebo, and patients previously randomized to placebo were assigned to receive oral linaclotide (290 μg) once a day. The combined FDA-recommended IBS-C responder endpoint was met by 33.6%, compared to 21% in the placebo group (*P* < 0.0001), and similarly a larger number of linaclotide-treated patients (50.1%) reported a reduction of ≥30% in abdominal pain, compared to the placebo group (37.5%; *P* = 0.0003). As in the 26 week clinical trial with linaclotide, improvement in abdominal pain was recorded within the first week of therapy and sustained throughout the treatment period. During the 4 week randomized withdrawal period, patients on linaclotide had continued relief of abdominal pain, while patients re-randomized to placebo showed a gradual worsening of abdominal pain symptoms to the level experienced by patients receiving placebo during the treatment period, however, without worsening of symptoms relative to baseline.

Finally, results from several pooled *post hoc* analyses of these two phase 3 trials further confirmed the efficacy of linaclotide treatment on symptoms of abdominal pain and discomfort in patients with IBS-C (Johnston et al., 2013). Importantly, it was shown in a *post hoc* longitudinal responder analysis using the FDA responder criterion that at week 3, more than 50% of linaclotide-treated patients reported >30% reduction in abdominal pain, and that this analgesic effect increased to greater than 60% of linaclotide-treated patients at week 7 and was sustained at approximately 70% of linaclotide-treated patients for the duration of the 26 week study (Castro et al., 2013).

Based on results from these two phase 3 pivotal trials, linaclotide received FDA approval in 2012 as a first-in-class drug for the treatment of adult patients with IBS-C, and by the European Medicines Agency for the symptomatic treatment of moderate to severe IBS-C in adult patients. Linaclotide was also approved in the US for the treatment of CIC (2012) and in Canada for the treatment of both IBS-C and CIC (2013). Today, linaclotide is in worldwide development for the treatment of IBS-C.

## SUMMARY AND PERSPECTIVE

This review offers a new perspective and understanding of endogenous mechanisms involved in the regulation of visceral pain. The intestinal GC-C/extracellular cGMP pathway is emerging as a novel pathway that regulates peripheral analgesia via inhibition of primary colonic afferents, which results in decreased visceral pain. Findings from both non-clinical models and clinical studies have confirmed that the analgesic effects of linaclotide are mediated by a distinct pain-regulating pathway that operates independently from improvements in bowel function. In order to maximize the benefits of these novel findings for patients work is ongoing to fully elucidate the underlying mechanisms of this pathway, such as the identification and characterization of the molecular target(s) on colonic afferents and secondly, to more broadly assess the significant therapeutic potential of targeting the GC-C/cGMP pathway in GI disorders associated with visceral pain. The efficacy of linaclotide on abdominal pain demonstrated in the phase 3 IBS-C clinical trials translates the targeting of the GC-C/cGMP pain pathway into the clinic. This pathway continues to emerge as a novel therapeutic target for GI dysfunction with the potential for broader utility in treating visceral pain.

## AUTHOR CONTRIBUTIONS

Alexander P. Bryant, Mark G. Currie, Gerhard Hannig, Caroline B. Kurtz, Inmaculada Silos-Santiago, and Boris Tchernychev contributed to writing and critical revisions of the manuscript.

## Conflict of Interest Statement

Alexander P. Bryant, Mark G. Currie, Gerhard Hannig, Caroline B. Kurtz, Inmaculada Silos-Santiago and Boris Tchernychev are employees and stock/stock option holders in Ironwood Pharmaceuticals, Inc.

## References

[B1] Bouin M. Plourde V. Boivin M. Riberdy M. Lupien F. Laganiere M. (2002). Rectal distention testing in patients with irritable bowel syndrome: sensitivity, specificity, and predictive values of pain sensory thresholds. *Gastroenterology* 122 1771–1777 10.1053/gast.2002.33601 12055583

[B2] Blackshaw L. A. Brierley S. M. (2013). Emerging receptor target in the pharmacotherapy of irritable bowel syndrome with constipation. *Expert Rev. Gastroenterol. Hepatol.* 7(Suppl. 1) 15–19 10.1586/17474124.2013.820045 23859756

[B3] Bryant A. P. Busby R. W. Bartolini W. P. Cordero E. A. Hannig G. Kessler M. M. (2010). Linaclotide is a potent and selective guanylate cyclase C agonist that elicits pharmacological effects locally in the gastrointestinal tract. *Life Sci.* 86 760–765 10.1016/j.lfs.2010.03.015 20307554

[B4] Busby R. W. Bryant A. P. Bartolini W. P. Cordero E. A. Hannig G. Kessler M. M. (2010). Linaclotide, through activation of guanylate cyclase C, acts locally in the gastrointestinal tract to elicit enhanced intestinal secretion and transit. *Eur. J. Pharmacol.* 649 328–335 10.1016/j.ejphar.2010.09.019 20863829

[B5] Busby R. W. Kessler M. M. Bartolini W. P. Bryant A. P. Hannig G. Higgins C. S. (2013). Pharmacologic properties, metabolism, and disposition of linaclotide, a novel therapeutic peptide approved for the treatment of irritable bowel syndrome with constipation and chronic idiopathic constipation. *J. Pharmacol. Exp. Ther.* 344 196–206 10.1124/jpet.112.199430 23090647

[B6] Carrithers S. L. Barber M. T. Biswas S. Parkinson S. J. Park P. K. Goldstein S. D. (1996). Guanylyl cyclase C is a selective marker for metastatic colorectal tumors in human extraintestinal tissues. *Proc. Natl. Acad. Sci. U.S.A.* 93 14827–14832 10.1073/pnas.93.25.14827 8962140 PMC26221

[B7] Castro J. Harrington A. M. Hughes P. A. Martin C. M. Ge P. Shea C. M. (2013). Linaclotide inhibits colonic nociceptors and relieves abdominal pain via guanylate cyclase-C and extracellular cyclic GMP. *Gastroenterology* 145 1334–1346 10.1053/j.gastro.2013.08.017 23958540

[B8] Cervetto C. Maura G. Marcoli M. (2010). Inhibition of presynaptic release-facilitatory kainate autoreceptors by extracellular cyclic GMP. *J. Pharmacol. Exp. Ther.* 332 210–219 10.1124/jpet.109.154955 19794031

[B9] Chey W. D. Lembo A. J. Lavins B. J. Shiff S. J. Kurtz C. B. Currie M. G. (2012). Linaclotide for irritable bowel syndrome with constipation: a 26-week, randomized, double-blind, placebo-controlled trial to evaluate efficacy and safety. *Am. J. Gastroenterol.* 107 1702–1712 10.1038/ajg.2012.254 22986437

[B10] Currie M. G. Fok K. F. Kato J. Moore R. J. Hamra F. K. Duffin K. L. (1992). Guanylin: an endogenous activator of intestinal guanylate cyclase. *Proc. Natl. Acad. Sci. U.S.A.* 89 947–951 10.1073/pnas.89.3.947 1346555 PMC48362

[B11] Drossman D. A. (2006). The functional gastrointestinal disorders and the Rome III process. *Gastroenterology* 130 1377–1390 10.1053/j.gastro.2006.03.008 16678553

[B12] Eutamene H. Bradesi S. Larauche M. Theodorou V. Beaufrand C. Ohning G. (2010). Guanylate cyclase C-mediated antinociceptive effects of linaclotide in rodent models of visceral pain. *Neurogastroenterol. Motil.* 22 312–e84 10.1111/j.1365-2982.2009.01385.x 19706070

[B13] Farrar J. T. Young J. P. LaMoreaux L. Werth J. L. Poole R. M. (2001). Clinical importance of changes in chronic pain intensity measured on an 11-point numerical pain rating scale. *Pain* 94 149–158 10.1016/S0304-3959(01)00349-9 11690728

[B14] Feng B. Gebhart G. F. (2011). Characterization of silent afferents in the pelvic and splanchnic innervations of the mouse colorectum. *Am. J. Physiol. Gastrointest. Liver Physiol.* 300 G170–G180 10.1152/ajpgi.00406.2010 21071510 PMC3025511

[B15] Feng B. Kiyatkin M. E. La J.-H. Ge P. Solinga R. Silos-Santiago I. (2013). Activation of guanylate cyclase-C attenuates stretch responses and sensitization of mouse colorectal afferents. *J. Neurosci.* 33 9831–9839 10.1523/JNEUROSCI.5114-12.2013 23739979 PMC3739058

[B16] Food and Drug Administration. (2010). Guidance for industry. Irritable bowel syndrome – clinical evaluation of products for treatment. Available at: http://www.fda.gov/Drugs/GuidanceComplianceRegulatoryInformation/Guidances/default.htm (accessed August 1, 2012).

[B17] Forte L. R. (1999). Guanylin regulatory peptides: structures, biological activities mediated by cyclic GMP and pathobiology. *Regul. Pept.* 81 25–39 10.1016/S0167-0115(99)00033-6 10395405

[B18] Hughes P. A. Brierley S. M. Martin C. M. Brookes S. J. Linden D. R. Blackshaw L. A. (2009). Post-inflammatory colonic afferent sensitization: different subtypes, different pathways and different time courses. *Gut* 58 1333–1341 10.1136/gut.2008.170811 19324867

[B19] Johnston J. M. Shiff S. J Quigley E. M. M. (2013). A review of the clinical efficacy of linaclotide in irritable bowel syndrome with constipation. *Curr. Med. Res. Opin.* 29 149–160 10.1185/03007995.2012.754743 23198977

[B20] Kita T. Smith C. E. Fok K. F. Duffin K. L. Moore W. M. Karabatsos P. J. (1994). Characterization of human uroguanylin: a member of the guanylin peptide family. *Am. J. Physiol.* 266 F342–F348 8141334 10.1152/ajprenal.1994.266.2.F342

[B21] Linden D. J. Dawson T. M. Dawson V. L. (1995). An evaluation of the nitric oxide/cGMP/cGMP-dependent protein kinase cascade in the induction of cerebellar long- term depression in culture. *J. Neurosci.* 15 5098–5105 7623138 10.1523/JNEUROSCI.15-07-05098.1995 PMC6577876

[B22] Lucas K. A. Pitari G. M. Kazerounian S. Ruiz-Stewart I. Park J. Schulz S. (2000). Guanylyl cyclases and signaling by cyclic GMP. *Pharmacol. Rev.* 52 375–413 10977868

[B23] Mann E. A. Jump M. L. Wu J. Yee E. Giannella R. A. (1997). Mice lacking the guanylate cyclase C receptor are resistant to STa-induced intestinal secretion. *Biochem. Biophys. Res. Commun.* 239 463–466 10.1006/bbrc.1997.7487 9344852

[B24] Marx U. C. Klodt J. Meyer M. Gerlach H. Roesch P. Forssmann W. G. (1998). One peptide, two topologies: structure and interconversion dynamics of human uroguanylin isomers. *J. Pept. Res.* 52 229–240 10.1111/j.1399-3011.1998.tb01480.x 9774236

[B25] Mertz H. Naliboff B. Munakata J. Niazi N. Mayer E. A. (1995). Altered rectal perception is a biological marker of patients with irritable bowel syndrome. *Gastroenterology* 109 40–52 10.1016/0016-5085(95)90267-8 7797041

[B26] Perkins A. Goy M. F. Li Z. (1997). Uroguanylin is expressed by enterochromaffin cells in the rat gastrointestinal tract. *Gastroenterology* 113 1007–1014 10.1016/S0016-5085(97)70198-7 9287996

[B27] Pfeifer A. Aszodi A. Seidler U. Ruth P. Hofmann F. Faessler R. (1996). Intestinal secretory defects and dwarfism in mice lacking cGMP-dependent protein kinase II. *Science* 274 2082–2085 10.1126/science.274.5295.2082 8953039

[B28] Poupoloupou C. Nowak L. M. (1998). Extracellular 3′,5′ cyclic guanosine monophosphate inhibits kainate-activated responses in cultured mouse cerebellar neurons. *J. Pharmacol. Exp. Ther.* 286 99–109 9655847

[B29] Qian X. Prabhakar S. Nandi A. Visweswariah S. S. Goy M. F. (2000). Expression of GC-C, a receptor-guanylate cyclase, and its endogenous ligands uroguanylin and guanylin along the rostrocaudal axis of the intestine. *Endocrinology* 141 3210–3224 10965892 10.1210/endo.141.9.7644

[B30] Rao S. Lembo A. J. Shiff S. J. Lavins B. J. Currie M. G. Jia X. D. (2012). A 12-week, randomized, controlled trial with a 4-week randomized withdrawal period to evaluate the efficacy and safety of linaclotide in irritable bowel syndrome with constipation. *Am. J. Gastroenterol.* 107 1714–1724 10.1038/ajg.2012.255 22986440 PMC3504311

[B31] Ritter C. A. Jedlitschky G. Meyer zu Schwabedissen H. Grube M. Köock K. Kroemer H. K. (2005). Cellular export of drugs and signaling molecules by the ATP-binding cassette transporters MRP4 (ABCC4) and MRP5 (ABCC5). *Drug Metab. Rev.* 37 253–278 10.1081/DMR-200047984 15747503

[B32] Sager G. (2004). Cyclic GMP transporters. *Neurochem. Int.* 45 865–873 10.1016/j.neuint.2004.03.017 15312981

[B33] Schlossman J. Feil R. Hofmann F. (2005). Insights into cGMP signaling derived from cGMP kinase knockout mice. *Front. Biosci.* 10: 1279–1289 10.2741/1618 15769624

[B34] Schulz S. Green C. K. Yuen P. S. Garbers D. L. (1990). Guanylyl cyclase is a heat- stable enterotoxin receptor. *Cell* 63 941–948 10.1016/0092-8674(90)90497-3 1701694

[B35] Schulz S. Lopez M. J. Kuhn M. Garbers D. L. (1997). Disruption of the guanylyl cyclase-C gene leads to a paradoxical phenotype of viable but heat-stable enterotoxin- resistant mice. *J. Clin. Invest.* 100 1590–1595 10.1172/JCI119683 9294128 PMC508341

[B36] Silos-Santiago I. Hannig G. Eutamene H. Ustinova E. E. Bernier S. G. Ge P. (2013). Gastrointestinal pain: Unraveling a novel endogenous pathway through uroguanylin/guanylate cyclase-C/cGMP activation. *Pain* 154 1820–1830 10.1016/j.pain.2013.05.044 23748116

[B37] Sindic A. Schlatter E. (2006). Cellular effects of guanylin and uroguanylin. *J. Am. Soc. Nephrol.* 17 607–616 10.1681/ASN.2005080818 16382016

[B38] Skelton N. J. Garcia K. C. Goeddel D. V. Quan C. Burnier J. P. (1994). Determination of the solution structure of the peptide hormone guanylin: observation of a novel form of topological stereoisomerism. *Biochemistry* 33 13581–13592 10.1021/bi00250a010 7947768

[B39] Spiegel B. Bolus R. Harris L. A. Lucak S. Naliboff B. Esrailian E. (2009). Measuring irritable bowel syndrome patient-reported outcomes with an abdominal pain numeric rating scale. *Aliment. Pharmacol. Ther.* 30 1159–1170 10.1111/j.1365-2036.2009.04144.x 19751360 PMC2793273

[B40] Tack J. Talley N. J. Camilleri M. Holtmann G. Hu P. Malagelada J. R. (2006). Functional gastroduodenal disorders. *Gastroenterology* 130 1466–1479 10.1053/j.gastro.2005.11.059 16678560

[B41] Vaandrager A. B. (2002). Structure and function of the heat-stable enterotoxin receptor/guanylyl cyclase C. *Mol. Cell. Biochem.* 230 73–83 10.1023/A:1014231722696 11952098

[B42] Videlock E. J. Chang L. (2007). Irritable bowel syndrome: current approach to symptoms, evaluation, and treatment. *Gastroenterol. Clin. North Am.* 36 665–685 10.1016/j.gtc.2007.07.002 17950443

[B43] Zimmermann C. Gutmann H. Hruz P. Gutzwiller J. P. Beglinger C. Drewe J. (2005). Mapping of multidrug resistance gene 1 and multidrug resistance-associated protein isoform 1 and 5 mRNA expression along the human intestinal tract. *Drug Metab. Dispos*. 33 219–224 10.1124/dmd.104.001354 15523049

